# Cues for odor naming affect performance and brain connectivity

**DOI:** 10.3389/fnhum.2025.1671670

**Published:** 2025-12-02

**Authors:** Eda Nur Capkan, Funda Yildirim

**Affiliations:** 1 Section of Cognition, Data and Education, University of Twente, Enschede, Netherlands; 2 Doctoral Program in Neuroscience, Yeditepe University, Istanbul, Türkiye

**Keywords:** odor naming, multisensory integration, color cue, lexical cue, electroencephalography, granger causality analysis

## Abstract

Human olfactory perception and naming represent a complex example of multisensory integration, with growing interest in how cues from different modalities affect olfactory recognition and naming. While studies show that visual cues may support odor naming performance, little is known about how cueing and multisensory integration in odor naming tasks influence neural mechanisms. This study examined the cognitive mechanisms underlying odor identification and the effect of two visual cue types—lexical and color—using behavioral and EEG methods. It also investigated the impact of hedonic ratings, Tip of the Nose phenomenon, familiarity, and subjective recall experiences on odor naming. Forty participants took part in an odor identification task using Sniffin’ Sticks. For each trial, an odorant was first presented, followed by either a visual cue (a color patch associated with the odor source) or a lexical cue (a word fragment). Participants were then asked to name the odor. To examine the neural mechanisms involved in cue-assisted odor identification, the time window during odor naming after the visual cue presentation was analyzed. Connectivity analysis and behavioral performance were assessed to evaluate the effectiveness of the different cue types in supporting identification. Behavioral findings showed that lexical cues improved identification accuracy. Furthermore, hedonic ratings, familiarity, and experiences related to the TON were found to significantly affect naming performance. Odor familiarity and liking levels affected both response accuracy and response time, with more familiar and liked odors being identified both more accurately and more quickly. Granger causality analysis revealed that the color cue condition exhibited more numerous and stronger network connections compared to the lexical cue condition. The lexical cue condition demonstrated more restricted network activation with fewer connections, utilizing focused frontal-temporal and frontal-parietal circuits. In both conditions, prefrontal regions served as strong control hubs, and language networks were preserved. However, additional frontal-occipital connections were observed in the color cue condition, in the form of interhemispheric coordination and visual system integration. The findings demonstrated that cross-modal odor naming utilizes different neural connections depending on cue type, with lexical cues showing more direct access to linguistic areas while color cues exhibit more complex connectivity patterns.

## 1 Introduction

The sense of smell plays a fundamental role not only in identifying food or detecting environmental threats but also in supporting motor and cognitive processes, regulating mood, and facilitating social interactions (Stevenson, 2009; Millot et al., 2002; Johnson, 2011; Seubert et al., 2009). Despite its multifaceted importance, identifying and naming odors is cognitively demanding and is often considered less accessible to consciousness than other sensory modalities.

Even odors that people are frequently exposed to in daily life can become challenging to identify when asked to name them, correctly identifying even less than 50% of common odors (Cain, 1979; Engen and Ross, 1973; Olofsson and Gottfried, 2015). In contrast, objects from familiar categories, such as fruits or vehicles, were named with over 90% accuracy. These rates are significantly lower than dominant sensory modalities such as vision and audition (Herz and Engen, 1996; Cain, 1979). This difficulty may partially stem from the infrequent labeling of olfactory stimuli in everyday language (Stevenson, 2009). Visual objects and colors are systematically categorized through their referential nomenclature. When we see a cup of coffee, we can identify it as a cup of coffee. In contrast, we usually describe smells with more ambiguous expressions such as “pleasant” or “peculiar.” Odors cannot establish consistent and strong connections with semantic memory (Yeshurun and Sobel, 2010). Rather, smell often triggers diffuse, affect-laden impressions rather than discrete object representations (Herz, 2003). Odor recognition and classification are predominantly mediated by hedonic valuation. Thus, hedonic judgments constitute critical components in olfactory processing (Barkat et al., 2003; Bensafi et al., 2002).

The process of naming odors into words becomes increasingly complex as it progresses through successive stages. In the first stage, an object-related perception of the odor is formed. In the second stage, lexical-semantic integration, this perception is matched with a word or concept. In the final stage, this match is expressed verbally. The limited interaction between the olfactory system and the language system contributes to naming difficulties at each of these stages (Olofsson and Gottfried, 2015).

Odor identification typically relies on contextual cues to help bridge the gap between perception and meaning. A name, category, or color alongside an odor can significantly enhance naming accuracy and confidence (Demattè et al., 2006; Gilbert et al., 1996). Color may activate semantic associations through cross-modal correspondences (Demattè et al., 2006; Zellner et al., 2008), while verbal cues (e.g., word labels or category reminders) may directly activate relevant semantic pathways (De Araujo et al., 2005; Herz, 2003; Majid et al., 2018; Vanek et al., 2021). These cues not only improve behavioral performance (Spence, 2020) but also provide theoretical frameworks to explain how olfactory cognitive processes are supported by external factors.

The process of odor naming can involve multiple distinct memory networks and neural pathways, allowing the brain to produce the same perceptual response through different routes (Kjelvik et al., 2012). The piriform cortex activates during both passive odor perception and active naming, often in concert with subcortical regions (Savic et al., 2000). The orbitofrontal cortex (OFC) emerges as a central hub that links olfactory signals with semantic content, while the temporal pole and inferior frontal gyrus support transitions from sensory representation to lexical labeling (Kjelvik et al., 2012). Functional and structural connectivity data report consistent interactions among these regions, with disruptions in temporal–frontal networks corresponding to deficits observed in clinical populations (Olofsson et al., 2013). Lexical and sensory cues modulate this network (Olofsson et al., 2014). When odor names or semantic labels are provided, enhanced activation is observed in frontal regions, shifting processing from predominantly sensory to semantic circuits (Royet et al., 1999). Visual cues, such as pictures of odor sources, activate visual cortex and parietal regions, reinforcing the multimodal integration observed in frontal areas (Kjelvik et al., 2012).

Predictive perceptual representations are established in the anterior temporal lobe prior to explicit odor naming, followed by habituation after semantic matching, with the timing and sequence of activation indicating dynamic interactions between sensory, semantic, and memory systems during odor identification (Kjelvik et al., 2012; Olofsson et al., 2014).

This study focuses on how two distinct types of visual cues, a fundamental low-level visual feature, color, and a complex high-level visual feature, text, differentially influence the odor naming and the neural networks associated with this process. Lexical cues, such as odor names or semantic labels, have been shown to enhance behavioral performance and preferentially activate neural regions associated with semantic processing and language, including the inferior frontal gyrus, orbitofrontal cortex, insula, and anterior cingulate cortex (Han et al., 2021; Olofsson et al., 2014; Tu et al., 2023) These cues appear to shift processing emphasis from sensory to semantic networks, especially when odors are ambiguous or unfamiliar, resulting in context-dependent modulation of hedonic and identification responses. In contrast, low-level visual cues such as color engage cross-modal sensory integration areas including the fusiform gyrus, visual cortex, and parietal regions, facilitating odor identification by enhancing sensory-perceptual associations (Kjelvik et al., 2012; Rekow et al., 2021). The combination of such visual and olfactory inputs leads to more rapid and accurate naming, with increased activation in transmodal hubs like the orbitofrontal cortex, anterior temporal lobe, and anterior cingulate cortex.

Together, these findings suggest that while lexical cues predominantly recruit higher-order semantic and language networks to guide odor naming, color cues primarily influence early sensory and perceptual processing stages, highlighting distinct yet complementary pathways through which different types of visual information shape olfactory perception and identification.

An odor’s familiarity is another crucial aspect that affects its naming (Frank et al., 2011). Familiar odors are generally named more accurately and evoke more stable, distributed neural responses (Eek et al., 2023; Hörberg et al., 2024; Zhang et al., 2023). Neuroimaging studies have revealed that familiar and unfamiliar odors activate different brain networks (Saive et al., 2014; Savic et al., 2000). Particularly, familiar odors create more intense activity in the hippocampus and parahippocampal gyrus regions (Kjelvik et al., 2012; Royet et al., 2011). Familiarity not only facilitates encoding and retrieval processes but also modulates the effectiveness of visual and lexical cues, potentially altering their behavioral and neural impact (Cecchetto et al., 2020; Herz, 1997; Sezille et al., 2014). Familiarity also affects speed in the odor naming process; response times are shorter for familiar odors (Frank et al., 2011).

The tip-of-the-nose (TON) phenomenon, analogous to tip-of-the-tongue states in verbal memory, frequently occurs during odor identification tasks and offers insight into the cognitive mechanisms underlying odor naming (Lawless and Engen, 1977).

This subjective state, where individuals recognize an odor but cannot retrieve its name, reflects a dissociation between perceptual recognition and lexical access in olfactory processing (Jönsson et al., 2005). Studies have shown that TON experiences often precede successful odor identification and are associated with partial semantic activation, suggesting that semantic and lexical representations of odors can be accessed independently (Jönsson et al., 2005). During TON states, participants often demonstrate semantic knowledge about an odor’s category or contextual associations while failing to recall the specific name. This pattern may be more common in olfaction than in vision due to weaker associations between odors and their linguistic labels. Research on odor naming has further revealed that although participants may recognize an odor as familiar and pleasant, they often struggle to retrieve the appropriate verbal label, indicating that semantic knowledge often precedes lexical retrieval and that these two representations may be partially independent in memory (Jönsson and Olsson, 2003).

Our primary goal in this study is to understand the cognitive processes underlying olfactory identification by examining how external contextual cues and individual differences shape odor naming abilities. We focus specifically on two distinct types of cues: sensory information provided through color and lexical information provided exclusively through letter prompts. Our study compared two types of semantically meaningful visual cues: written lexical cues (letter fragments of odor names) and color cues (colors associated with odor sources). Both cue types were designed to be semantically relevant to the target odors, rather than comparing meaningful versus meaningless stimuli.

By analyzing participants’ naming accuracy across different cue conditions, we seek to clarify the contributions that color and lexical information make in supporting olfactory naming. Our analysis extends beyond basic performance metrics to include several key factors that may influence odor naming. We assess how odor familiarity, as measured through post-experiment questions, and response times affect naming latency. This relationship may reveal important insights into how semantic memory access facilitates or constrains olfactory recognition processes. Our investigation focuses on the TON experience; we hypothesize that TON states may indicate partial but incomplete access to odor representations, and we examine how these experiences relate to overall naming accuracy. Finally, our study explores the underlying neural mechanisms of odor naming by mapping connectivity patterns and investigating how different brain regions interact during olfactory processing, particularly in response to lexical versus color cues.

## 2 Materials and methods

### 2.1 Participants

The study recruited 40 Turkish-speaking volunteers, aged 18–32. The average age of the participants was 23.35 (SD = 3.31), and 65% of them were female. All participants reported a functioning sense of smell and vision, no respiratory tract diseases, were non-smokers, and were non-allergenic. They had no history of neurological and/or psychiatric diagnoses and were not taking any medication. All participants were self-reported non-smokers, had normal vision, no neurological and/or psychiatric diagnoses, and no reported olfactory dysfunction.

Participants who had refrained from consuming food, coffee, and tobacco products at least 2 hours before the experiment were included. They also did not use scent-containing products such as perfume.

Participants were recruited via social media platforms and the university’s student information system mailing list. An informative post was prepared, outlining the study’s purpose, EEG procedures, inclusion and exclusion criteria, and participation details. Interested participants selected a time slot for the experiment via Doodle. They also received written instructions, which asked them to avoid eating or drinking anything for at least 2 h prior to the session, and to refrain from using scented cosmetic products such as perfumes, deodorants, or lotions on the day of the experiment.

Before the experiment, all volunteers signed an informed consent form and received detailed explanations about the experimental procedures, both in writing and orally. Participation was entirely voluntary, and no incentives or rewards were offered for their participation.

The study protocol was approved by the Yeditepe University Ethical Committee for Social Sciences (approval number: E.50532705-604.02-5165) and agreed with the Declaration of Helsinki.

### 2.2 Stimuli and design

#### 2.2.1 Determination of odor stimuli

A preliminary investigation was conducted to determine the most suitable olfactory stimuli for assessing odor naming performance. Odor stimuli were selected from the standardized Sniffin’ Sticks set (Burghart Messtechnik, Wedel, Germany), which is widely used in olfactory research. The 31 odor stick pretest was conducted on 15 individuals (not included in the main experiment sample) to assess consistency in naming. The licorice odor was excluded prior to testing because it is not commonly encountered in the Turkish cultural context and was therefore considered likely to result in recognition bias.

Each odor was presented by positioning the pen approximately 2 cm below the participant’s nostrils, held centrally, and allowing a single sniff lasting around 2 s. Participants were asked to sniff once and verbally name the odor as accurately as possible. To prevent olfactory fatigue and cross-adaptation, a 20-s interval was maintained between each odor presentation. Furthermore, after every eight odors, participants were given a 1-min rest period.

Based on the analysis, 12 odors with different levels of naming accuracy were chosen for inclusion in the main study (see section 2.5.1). These odors were subsequently divided into two balanced groups for use in separate experimental conditions. Group 1 consisted of *Coconut, Anise, Lemon, Lavender, Mushroom* and *Grass*, while Group 2 consisted of C*aramel, Cola, Banana, Eucalyptus, Onion* and *Rose.*

This selection aimed to ensure a diverse, yet culturally relevant odor set, with moderate recognition difficulty in avoiding ceiling or floor effects in odor naming performance.

#### 2.2.2 Determination of visual stimuli: color and lexical cues

The visual stimuli used in the experiment were designed to provide either color-based or lexical (letter-based) cues associated with the odor sources. A systematic method was followed to determine an appropriate visual representation for each odor.

For the color cues, the representative colors of each odor source were identified through an image-based content analysis. First, the name of each odor (e.g., “lemon,” “caramel,” and “lavender”) was searched using Google Images. From the resulting images, those most frequently and clearly representing the typical or natural appearance of the odor source were selected. The primary colors in these images were derived using the Pantone Studio application. This application allowed for direct color sampling from images and provided the RGB (Red, Green, Blue) codes of the selected areas. For each odor, the two most frequently encountered and visually dominant colors were identified and selected as color cues to be used in the experiment. These colors were then used in the visual stimuli to provide perceptual hints associated with the odor.

**FIGURE 1:**
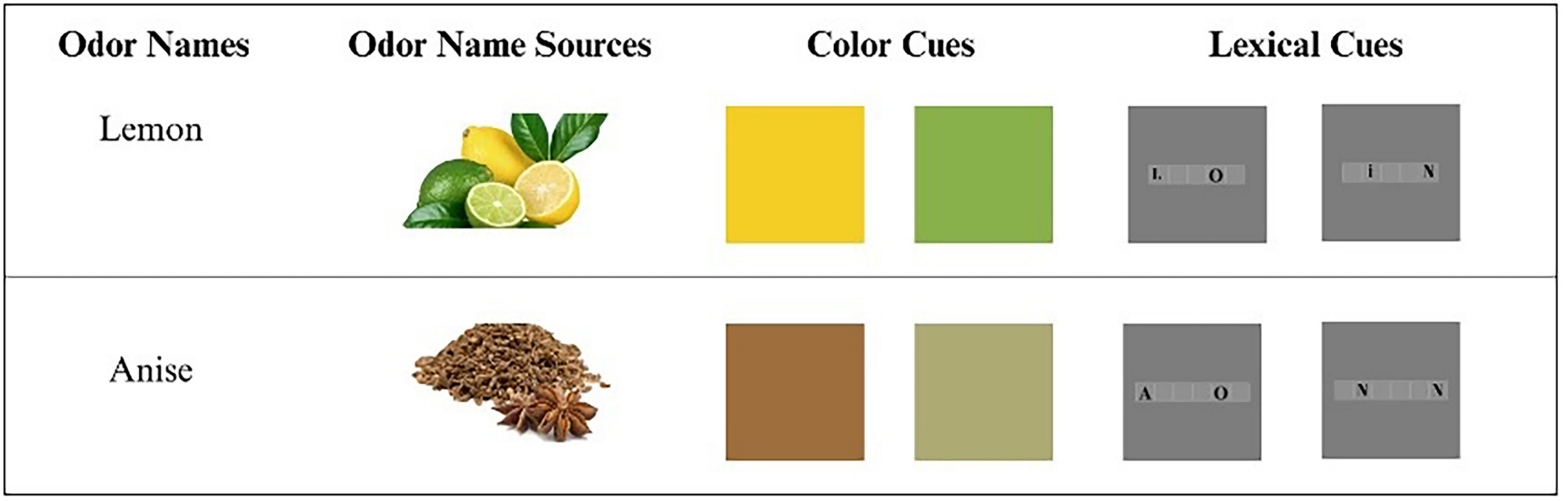
Odor source and cue types example. All lexical cues are presented in Turkish (e.g., lemon: *limon*, anise: *anason*). Adapted from Canva content, used under Canva’s Content License Agreement.

For the lexical cues, a minimal linguistic representation derived from each odor’s name was designed. Each lexical cue consisted of two letters from the name of the odor (e.g., “L- - - O -” and “- E - - - N” for lemon, “B - - - N -” and “- A - - - A” for banana). Care was taken to ensure that the selected letter pairs were legible and visually consistent across stimuli. The purpose of the lexical cue was to activate semantic memory related to the odor source without directly revealing the odor’s full name, thus providing only a subtle associative hint (see Figure 1). The lexical and color cues were determined for each odor and presented to an independent group of 15 participants, who were not part of the main sample. In this pretest, the consistency of cue naming was evaluated based on the participants’ responses. Naming consistency was defined as the proportion of participants who provided the correct odor label.

All visual stimuli were presented against the same background color to ensure consistency across conditions. To control for potential luminance differences for visual cues that could influence visual attention, the luminance of all stimuli was normalized using the SHINE toolbox (Willenbockel et al., 2010) This procedure ensured that any observed effects could be attributed to the cue type rather than variations in brightness or contrast between stimuli.

As a result, two distinct visual stimuli were developed for each odor, two for the color cue condition and two for the lexical cue condition (see Figure 1 for an example). These stimuli were used across different experimental conditions to investigate the effects of visual cue type on odor naming performance.

### 2.3 Electrophysiological recording

EEG data were recorded using a BrainAmp EEG amplifier, Brain Vision Recorder software (BrainProducts, Munich, Germany), and a BrainCap electrode cap with 32 channels. Electrodes were placed according to the international 10–20 system at standard scalp positions, and recordings were obtained from the following sites: Fp1, Fp2, F7, F3, Fz, F4, F8, FT7, FC3, FCz, FC4, FT8, Cz, C3, C4, T7, T8, TP7, CP3, CPz, CP4, TP8, TP9, TP10 P3, Pz, P4, P7, P8, O1, Oz, and O2. Conductive electrolyte gel was applied between the scalp and the electrodes.

Two linked earlobe electrodes (A1 + A2) were used as reference electrodes. All electrodes were made of Ag/AgCl. EEG signals were digitized online at a sampling rate of 500 Hz. Recordings were conducted in a dimly lit, sound- and light-attenuated, electrically shielded room.

### 2.4 Experimental procedure

The entire experiment, including instructions and all stimulus presentations, was conducted in Turkish to ensure that participants fully understood the instructions and provided natural responses.

Before the main experiment began, participants took part in practice sessions consisting of two odors each. The odors and colors presented in these practice sessions did not overlap with those used in the main experiment. After the practice session, the main experiment commenced. Participants were provided verbal information before the experiment and an informative text about the general flow of the experiment during the session. Participants began the experiment by pressing any key on the keyboard once they felt ready.

Before each odor presentation, a fixation mark (“+”) was shown at the center of the screen for 5 s. Then, the command “Smell” appeared on the screen, signaling that the odor presentation was about to begin. The odor stick was held 2–4 cm away from the participant’s nostrils, and participants were instructed to smell the odor with their eyes open, taking a single breath. This odor presentation process lasted for 2.5 s.

**FIGURE 2:**
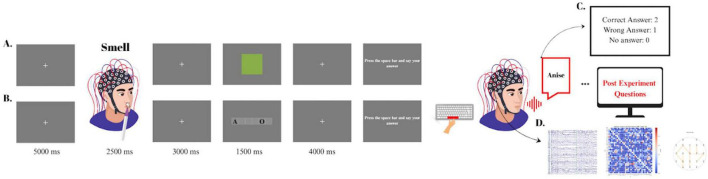
Experimental design and data processing pipeline. **(A)** Color cue condition timeline: fixation (5,000 ms) → smell the odor (2,500 ms) → fixation (3,000 ms) → color cue (1,500 ms) → fixation (4,000 ms) →response prompt→ post-experimental questions. **(B)** Lexical cue condition with identical timing but letter fragment cues, followed by post-experimental questions. **(C)** Behavioral coding scheme: responses categorized as correct (2), incorrect (1), or no answer (0). **(D)** EEG preprocessing workflow including artifact removal, Granger causality connectivity matrix generation, and 3D head model visualization for network analysis.

After the odor presentation, a 3-s fixation mark was displayed, followed by a 1.5-s visual stimulus. A 4-s waiting period was then provided to allow participants to process the odor-cue pairing and formulate their response. Following this thinking period, the instruction “Press the spacebar and then provide your answer verbally. Then say your answer” appeared on the screen. Response time (RT) was measured as the interval between the appearance of this response prompt and the participant’s spacebar press, reflecting the time needed to finalize their decision and initiate response. Participants pressed the space bar and then verbally provided their response regarding the odor (see Figure 2).

The 12 odors were divided into two balanced groups for counterbalancing purposes. Participants were randomly assigned to one of two counterbalanced conditions: half of participants (*n* = 20) were randomly assigned to experience Group 1 odors (Coconut, Anise, Lemon, Lavender, Mushroom, Grass) with lexical cues and Group 2 odors (Caramel, Cola, Banana, Eucalyptus, Onion, Rose) with color cues, while the other half (*n* = 20) were randomly assigned to the reverse pairing. This randomization and counterbalancing ensured that each odor was tested equally often with both cue types across participants, controlling for odor-specific effects. In total, each participant experienced 6 repetitions of each odor under their assigned cue condition, resulting in 6 odors × 6 repetitions = 36 trials per cue type, for a total of 72 trials per participant (2 × 36 trials). The experiment was conducted in two separate blocks, with 36 trials in each block (one block for color cues, one block for lexical cues). A 20-min break was given between the two cue type sessions.

The stimuli were presented on an HP x22LED 21.5-inch LED backlit LCD monitor with a 16:9 aspect ratio, 1920 × 1080 resolution, and 60 Hz refresh rate.

The experimental task was designed using MATLAB, and the stimuli were presented using Psychophysics Toolbox (Psychtoolbox-3) (Brainard, 1997; Kleiner et al., 2007; Pelli, 1997).

Once all trials were completed, a screen indicated that the experiment had ended. Following this, participants were asked to answer a series of questions based on the study by Jönsson et al. (2005) (see Supplementary material). These questions aimed to assess participants’ “Tip of the Nose” (TON) experience and subjective evaluation of odor preferences.

During the experiment, participants were seated 60 cm away from the screen where stimuli were presented. The computer screen used in the experiment was aligned with the participant’s eye level. EEG activity was recorded throughout the experimental session.

### 2.5 Data analysis

#### 2.5.1 Determination of odor stimuli

The K-means clustering analysis was applied to classify odors based on their naming accuracy. In this analysis, the average (M), coefficient of variation (CV), and total score (TS) values were considered for each odor. The number of clusters was set to *k* = 3, and accordingly, the odors were divided into three groups with high, medium, and low accuracy levels. According to the clustering analysis, we selected four odors with high identification, four odors with medium identification, and four odors with low identification.

In the high accuracy group, odors with high recognition accuracy are included. The odors in this group are eucalyptus (TS = 6.6, M = 0.44, CV = 0.67), caramel (TS = 6.4, M = 0.427, CV = 1.14), anise (TS = 5.8, M = 0.387, CV = 1.101), and coconut (TS = 5.6, M = 0.373, CV = 1.17). The medium accuracy group contains odors with moderate recognition accuracy. This group includes lemon (TS = 4.3, M = 0.287, CV = 1.159), banana (TS = 4.3, M = 0.287, CV = 1.413), grass (TS = 4.4, M = 0.293, CV = 1.522), and rose (TS = 4.0, M = 0.267, CV = 1.563). The low accuracy group consists of odors with low recognition accuracy. These odors are onion (TS = 3.7, M = 0.247, CV = 1.412), lavender (TS = 3.4, M = 0.227, CV = 1.776), mushroom (TS = 3.2, M = 0.213, CV = 1.915), and cola (TS = 2.1, M = 0.140, CV = 2.501).

#### 2.5.2 Behavioral data

Participants’ responses in the odor naming task were categorized as correct, incorrect, or no answer. To examine the association between odor naming accuracy and the cue condition (visual or lexical), a Chi-squared test of independence was conducted. However, the Chi-squared test does not account for the hierarchical structure of the data, such as repeated measurements within participants and dependencies among odor items. To address these dependencies and obtain more reliable estimates, we additionally fitted generalized linear mixed-effects models (GLMM) with logit link functions, including cue type as a fixed effect and participants and odors as random effects.

To investigate the effect of cue types on response time, differences between the two independent groups were analyzed. As the data did not meet the assumptions of normality, the Mann-Whitney U test was performed.

Participants rated their familiarity with the odors, liking, and the TON feeling using a 9-point Likert scale. Given the ordinal nature of these variables, Spearman’s rank-order correlation and Kendall’s tau-b analyses were conducted to assess their relationship with odor naming response time and accuracy.

A Kruskal-Wallis test was conducted to examine the effect of familiarity and liking levels on response accuracy in the odor naming task. To identify specific group differences, Dunn’s *post hoc* test with the Bonferroni correction was applied. To further explore the effect of the TON feeling on response time, the Kruskal–Wallis H test was applied when comparisons among more than two groups were required. When appropriate, non-parametric correlation analyses (Spearman and Kendall) were conducted to support the findings and evaluate the strength and direction of associations among variables.

All statistical analyses were performed using JASP (version 0.19.3) and the Python programing environment, accessed through the Anaconda distribution with a Jupyter Notebook interface (version 7.2.2). Visualizations were created using the same Jupyter Notebook interface. A significance level of *p* < 0.05 was used in all tests.

#### 2.5.3 EEG data

##### 2.5.3.1 Preprocessing of EEG data

Matlab (version R2024b) was used to process and analyze the olfactory EEG, and the EEGLAB (version 2025.0.0) and were Jupyter Notebook (version 7.2.2) used for preprocessing. The EEG preprocessing steps began with loading the raw data. The sampling rate was maintained at 500 Hz without any downsampling. First, the signals were filtered using a Finite Impulse Response (FIR) filter (0.1–48 Hz). After filtering, bad channels caused by poor contact were identified and repaired using global spline interpolation to maintain data quality (Guo et al., 2024). To improve the accuracy of the EEG data, a common average reference (CAR) was used as the average reference. This method ensured that each channel’s data was referenced to the mean of all channels’ data, providing a more balanced dataset (Lepage et al., 2014). Artifacts, particularly those from eye blinks, saccades, and muscle activity, were removed using Fast Independent Component Analysis (Chiarion et al., 2023). The dataset was then segmented into epochs corresponding to each event.

Signal loss occurred in the Oz channel (occipital region, posterior midline electrode in the 10-20 system) in the first seven participants due to technical malfunctions. In this case, the spherical spline interpolation method was applied to recover the missing channel data (Perrin et al., 1989).

##### 2.5.3.2 Granger causality analysis for EEG connectivity

To investigate directed information flow between brain regions during olfactory processing under lexical and color cue conditions, we employed Granger causality analysis on EEG time-series data. Granger causality provides a statistical framework for assessing whether the inclusion of past values from one signal improves the prediction of another, thereby revealing the direction of information flow (Granger, 1969; Seth et al., 2015).

This method is particularly well-suited for M/EEG data due to its high temporal resolution, which enables the tracking of rapid neural interactions. In contrast to undirected connectivity measures, Granger causality allows for the dissociation of X→Y from Y→X, making it valuable for comparing experimental conditions in terms of directionality (Barrett et al., 2012; Casile et al., 2021).

We specifically employed conditional Granger causality, which offers improved sensitivity in distinguishing direct from indirect influences and demonstrates reliable performance across various neurophysiological data contexts with relatively low computational cost (Blinowska, 2011). To examine the bidirectional information flow between brain regions during olfactory naming processing under different cueing conditions, we employed Granger causality analysis rather than traditional ERP or correlation-based approaches. This choice was motivated by our central research question, which is to determine whether lexical and color cues engage distinct neural network organizations and to identify which brain regions drive connectivity to others. Unlike ERP or correlation methods, Granger causality enables the assessment of directed connectivity, providing insights into the directionality of information transfer rather than merely activation levels or undirected associations. The analysis was implemented using a custom pipeline in Jupyter Notebook (v7.2.2), which incorporated essential preprocessing steps, including bandpass filtering, epoch segmentation, and testing for covariance stationarity. Time-series modeling was performed using Vector Autoregressive (VAR) models, which estimate the joint temporal dynamics of multivariate signals (Cohen, 2014).

This approach allowed us to identify directed functional connectivity patterns modulated by cue type, offering insights into the temporal organization of neural processing during multisensory integration.

###### 2.5.3.2.1 Stationarity assessment and preprocessing

A critical prerequisite for valid Granger causality analysis is the stationarity of input time series, as non-stationary data can lead to spurious regression results and unreliable causal inferences (Seth et al., 2015). Therefore, the Dickey-Fuller test was applied to assess stationarity of each EEG channel within each experimental epoch (Dickey and Fuller, 1979).

A systematic differencing procedure was implemented for channels exhibiting non-stationarity. First-order differencing was applied initially, followed by re-testing for stationarity. If required, second-order differencing was employed, with a maximum of two differencing operations to preserve signal interpretability while achieving stationarity (Barnett and Seth, 2014). This approach strikes a balance between the need for stationarity and the preservation of meaningful temporal dynamics in the neural signals.

###### 2.5.3.2.2 Epoch-wise analysis framework

The analysis was conducted on an epoch-by-epoch basis to account for the temporal dynamics and potential non-stationarity inherent in task-related EEG data. Each experimental epoch was considered an independent realization of the underlying neural process, consistent with established practices in EEG connectivity analysis (Barrett et al., 2012). This approach provides several methodological advantages. It accommodates trial-to-trial variability in neural responses, enables the evaluation of consistency in connectivity patterns across experimental conditions, and offers strong statistical inference through repeated sampling across epochs.

###### 2.5.3.2.3 Model order selection

The selection of optimal lag order (p) in the VAR model represents a crucial balance between model complexity and predictive accuracy. To determine the appropriate lag length for the model, model selection criteria such as the Akaike Information Criterion (AIC), Hannan-Quinn Information Criterion (HQC), and Bayesian Information Criterion (BIC) were used (Akaike, 1974; Biagi and Pulina, 2009; Schwarz, 1978). Following established recommendations for EEG data analysis, a maximum lag order of 10 was implemented, corresponding to an approximate time delay of 20 milliseconds.

###### 2.5.3.2.4 Data quality control and outlier adjustment

Prior to group-level analysis, connectivity estimates were subjected to quality control procedures to address potential artifacts. Individual F-statistics derived from Granger causality tests were examined for outliers using the interquartile range (IQR) method. Outliers were defined as observations exceeding the third quartile (Q3) + 1.5 × IQR or falling below the first quartile (Q1) − 1.5 × IQR. To preserve statistical power while limiting the influence of extreme values, the winsorizing technique was applied: outlying F-values were capped at the computed thresholds rather than removed entirely (Bahadori and Liu, 2013; Utoft et al., 2024). This approach retains the relative ranking of connectivity strengths while preventing extreme values from disproportionately affecting group-level statistics (Blaine, 2018). As a result of this correction procedure, the full dataset from 40 participants was retained for group analysis, thereby maximizing statistical power.

###### 2.5.3.2.5 Multi-subject group analysis

To establish population-level patterns of directed connectivity, individual subject results were aggregated using a comprehensive statistical framework. For each channel pair, the consistency of Granger causal relationships across subjects was quantified by calculating the proportion of subjects exhibiting statistically significant causality (*p* < 0.05). Group-level significance was assessed using a threshold criterion: connections present in more than 95% of subjects were deemed highly reliable patterns. This threshold approach enables the identification of both robust core connectivity patterns and more variable subject-specific relationships.

###### 2.5.3.2.6 Statistical inference and multiple comparisons

Given the large number of pairwise connectivity tests inherent in multichannel EEG analysis, it is essential to have an appropriate control for multiple comparisons. The analysis framework calculated F-statistics and corresponding p-values for each directed connection. Multiple comparisons were controlled using the Benjamini-Hochberg false discovery rate (FDR) procedure, with statistical significance assessed at α = 0.05. To enhance interpretability, effect sizes were standardized using a standard deviation and z-score transformation of F-values across all tested connections.

The interpretation of Granger causality results requires careful consideration of the method’s limitations. As emphasized in the literature, Granger causality reflects predictive relationships rather than true causal mechanisms, and results should be interpreted as evidence of directed functional connectivity instead of direct neural causation (Seth et al., 2015; Shojaie and Fox, 2022).

###### 2.5.3.2.7 Visualization and interpretation framework

Granger causality results were visualized using multiple complementary approaches to capture the complexity of directed functional connectivity patterns. *F*-values from Granger causality analysis were displayed in connectivity heatmaps, with electrode pairs arranged according to the standard 10-20 EEG positioning system. Channel connections that showed significance in 95% of participants were marked with asterisks (*) to indicate high reliability across the sample. To identify the most robust connectivity patterns, threshold values were determined based on the distribution of F-values from both lexical and color cue conditions. Supra-threshold connections were projected onto a standardized brain model with electrode locations grouped into anatomical regions to enable interpretation of connectivity patterns within their functional neuroanatomical context. Differential connectivity patterns between experimental conditions were examined by computing difference heatmaps. The lexical condition heatmap was subtracted from the color cue condition heatmap to identify condition-specific connectivity differences. Connections showing significance in 95% of participants in the difference map were marked with asterisks to highlight reliable condition-related changes.

## 3 Results

### 3.1 Accuracy of odor naming response

**FIGURE 3:**
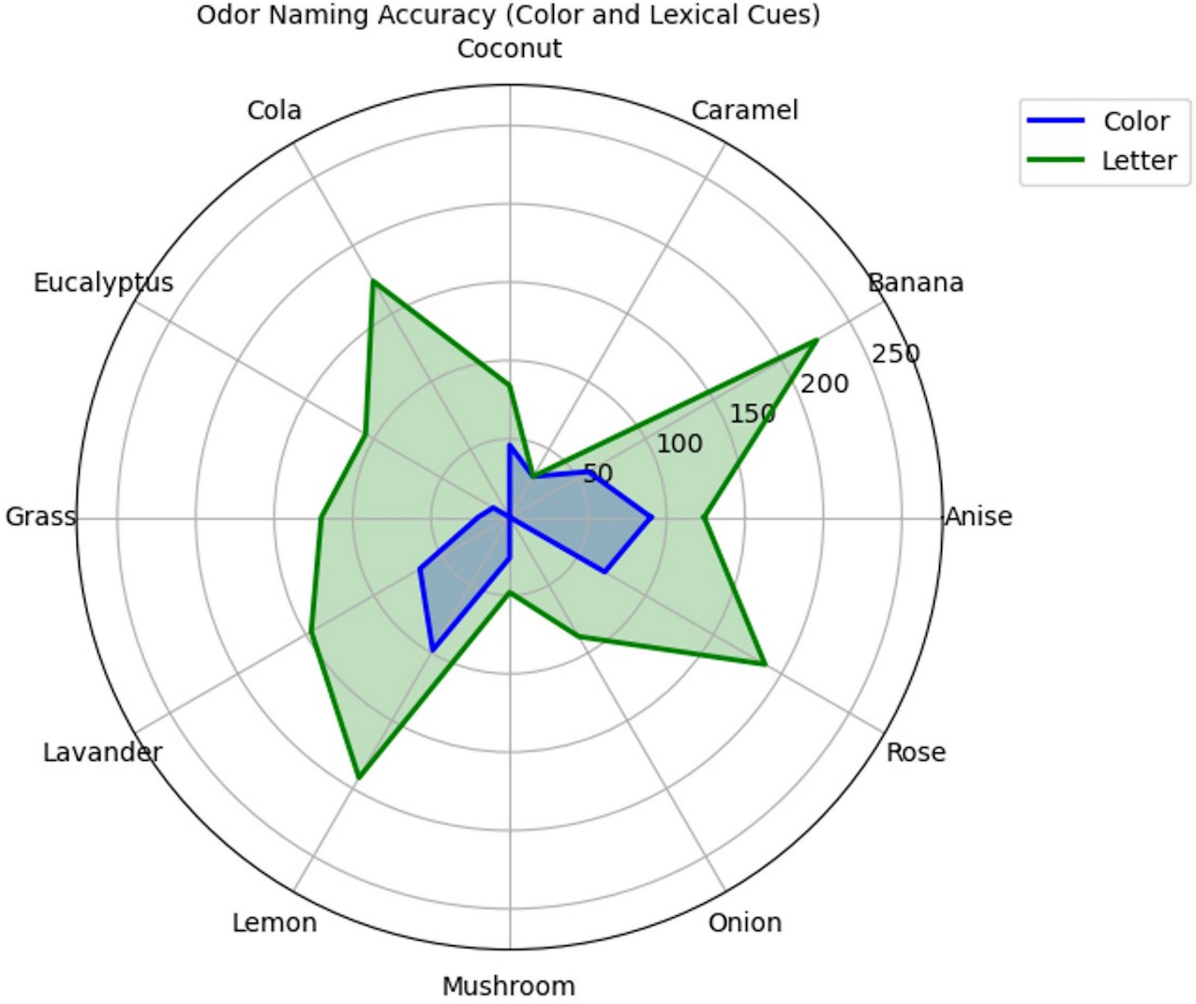
Odor naming accuracy under different cue conditions. Spider plot showing correct identification counts for 12 odors under color (blue) versus lexical (green) cue conditions. Radial distance indicates number of correct responses out of 40 participants per condition.

A total of 40 participants named odors under the color and lexical cue conditions (see Figure 3). The analysis shows that under the lexical cue, banana odor had the highest correct naming count, with 113 correct names, followed by lemon odor with 96 correct names. Conversely, under the color cue, lemon and anise odor demonstrated the highest correct naming counts, with 49 and 45 correct names, respectively. In contrast, cola and onion odor had the lowest correct naming counts under the color cue, with zero correct names.

A total of 258 correct names were made under the color cue condition, while 763 correct names were made under the lexical cue condition (Table 1).

**TABLE 1:** Contingency table of odor naming accuracy by cue condition.

	Accuracy			
Condition type	No answer	Incorrect answer	Correct answer	Total
Color	442	740	258	1,440
Lexical	439	238	763	1,440
Total	881	978	1,021	2,880

Each cell displays the observed counts.

A chi-squared test was conducted to examine the association between odor naming accuracy (categorized as correct, incorrect, and no answer) and cue condition (color and lexical). Participants showed significantly higher odor naming accuracy with lexical cues compared to color cues, as evidenced by a significant association between cue type and naming performance (χ^2^ = 507.463, *p* < 0.001, df = 2, *N* = 2,880, chi-square test, two-tailed).

To examine the effect of cue type on odor naming performance while accounting for dependencies due to repeated measurements within participants and odor-specific effects, we employed generalized linear mixed-effects models with logit link functions. The hierarchical structure of our data (2,880 trials from 40 participants × 12 odors × 6 trials each) necessitated mixed-effects modeling to properly control for participant and odor variability.

Two separate GLMMs were fitted. The first model investigated the probability of providing any response. In this model, cue type was included as a fixed effect, while participants and odors were included as random intercept effects. The second model examined accuracy among those trials where a response was given, again with cue type as a fixed effect and participants and odors as random intercept effects.

The first model investigated whether participants provided any response (combining incorrect and correct responses vs. no response). Results revealed no significant difference in response probability between cue conditions (β = 0.008, SE = 0.092, *z* = 0.09, *p* = 0.928). The estimated marginal probability of providing any response was 0.764 (95% CI [0.642, 0.853]) for lexical cues and 0.762 (95% CI [0.641, 0.852]) for color cues. The odds ratio comparing lexical to color cues was 1.008 (95% CI [0.841, 1.209]).

The second model examined accuracy among participants who provided responses (*N* = 1,999 trials), comparing correct versus incorrect responses. This analysis revealed a highly significant effect of cue type on naming accuracy (β = 2.894, SE = 0.141, *z* = 20.48, *p* < 0.001). The estimated marginal probability of correct identification was 0.805 (95% CI [0.670, 0.893]) for lexical cues versus 0.186 (95% CI [0.101, 0.317]) for color cues. Lexical cues were associated with 18.07 times higher odds of correct identification compared to color cues (95% CI [13.70, 23.84]).

These findings demonstrate that while both cue types were equally effective at prompting response attempts, lexical cues provided substantially greater support for accurate odor naming.

### 3.2 Influence of cue types on response time in odor naming

To investigate whether different cue types influence the response time of odor naming, the Mann-Whitney U test was used to examine the differences between the color and lexical cue conditions. Participants showed similar response times for odor naming regardless of cue type, with color cues yielding mean response times of 2.301 s (SD = 3.605) and lexical cues 2.271 s (SD = 3.521) (U = 1.038 × 10^6^, *p* = 0.948, N_color_cue_ = 1,440, N_lexical_cue_ = 1,440, two-tailed Mann-Whitney U test).

### 3.3 Influence of odor familiarity and liking on odor naming accuracy

**FIGURE 4:**
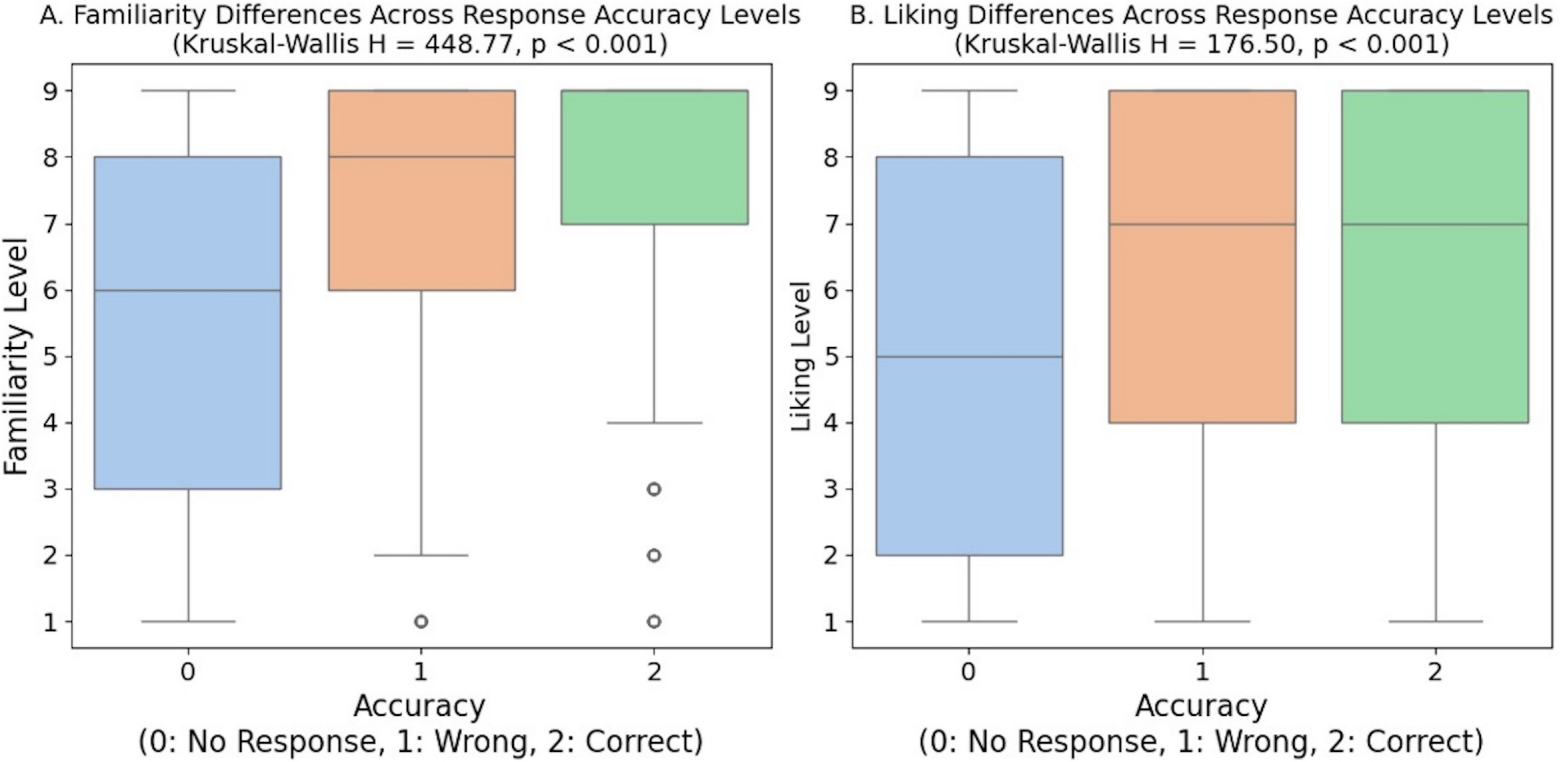
Odor familiarity and liking effects on naming accuracy. Box plots showing **(A)** familiarity ratings (1–9 scale) and **(B)** hedonic liking ratings (1–9 scale) across response accuracy categories (0 = no response, 1 = incorrect, 2 = correct). Higher familiarity and liking scores correlate with increased naming accuracy. Kruskal-Wallis tests revealed significant differences between all groups (*p* < 0.001).

To examine the effect of familiarity and liking levels on response accuracy in an odor naming task, a Kruskal-Wallis test was conducted. Participants who provided correct naming responses showed significantly higher familiarity and liking ratings compared to those who gave incorrect naming responses or no responses, demonstrating a clear relationship between subjective odor experience and naming accuracy (Figure 4). Specifically, familiarity levels differed significantly across response accuracy groups (no response, incorrect response, correct response) (*H* = 448.77, *p* < 0.001, df = 2, *N* = 2,880, Kruskal-Wallis test). Similarly, liking levels showed significant differences across the three groups (*H* = 176.50, *p* < 0.001, df = 2, *N* = 2,880, Kruskal-Wallis test). *Post hoc* Dunn’s tests with Bonferroni correction revealed significant differences between all pairwise group comparisons (all *p* < 0.05).

### 3.4 Influence of odor familiarity and liking on response time in odor naming

**FIGURE 5:**
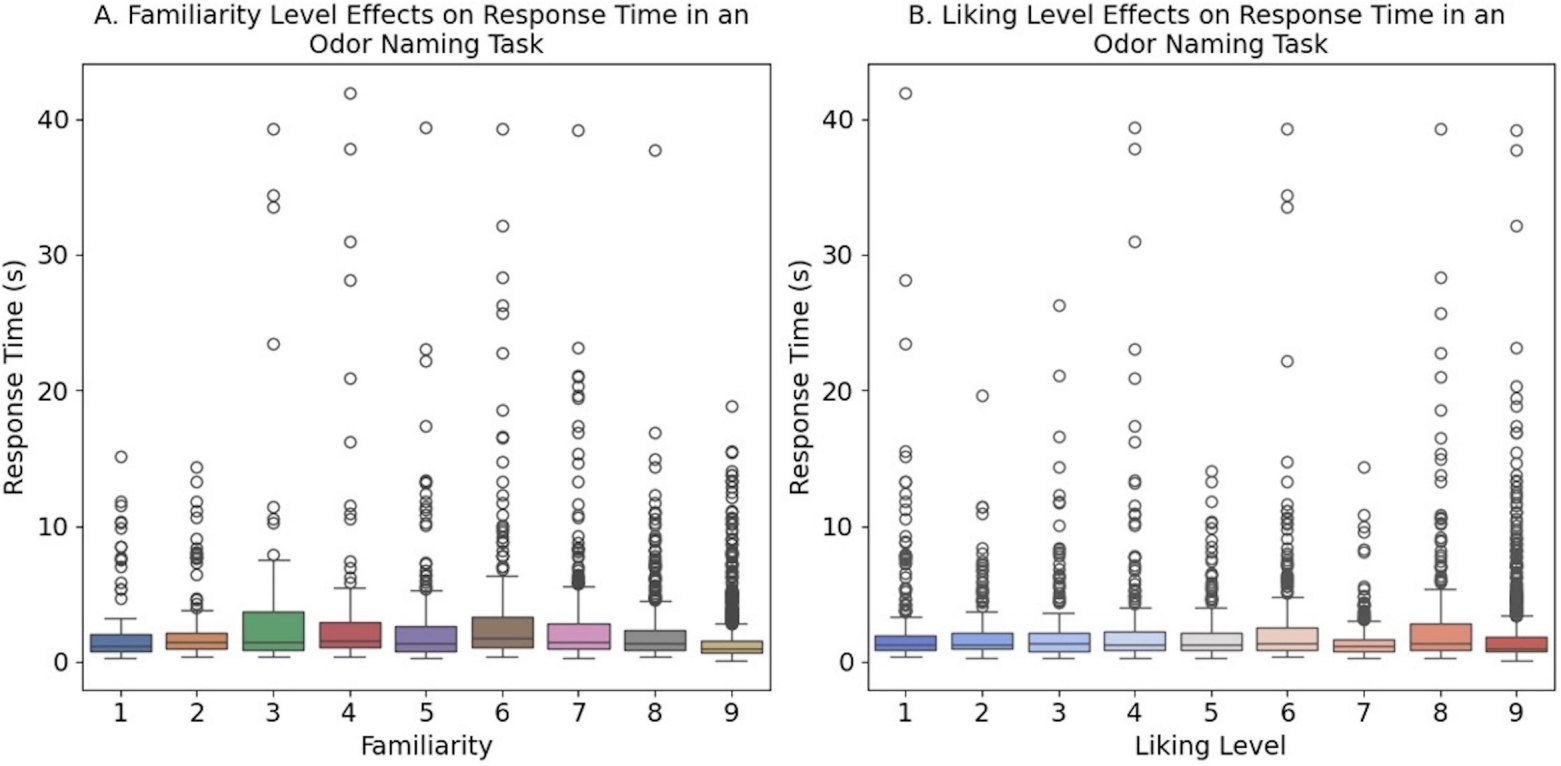
Correlation between odor properties and response time. *Scatter plots with box plots* showing relationships between **(A)** odor familiarity level (1–9 scale) and response time, and **(B)** odor liking level (1–9 scale) and response time. Both familiarity (ρ = - 0.211, *p* < 0.001) and liking (ρ = - 0.081, *p* < 0.001).

To examine whether participants’ subjective ratings of odor familiarity and liking affect the response time of odor naming, we conducted Spearman and Kendall correlation analyses between these ratings and response times. Participants took significantly longer to name less familiar odors, with response times decreasing from 3.440 s for low familiarity odors (level 3) to 1.602 s for highly familiar odors (level 9), demonstrating that more familiar odors were named more quickly (Figure 5) (ρ = −0.211, *p* < 0.001, *N* = 2,880, two-tailed Spearman rank correlation). This moderate negative relationship between familiarity and naming response time was consistently observed across different correlation methods (τB = −0.154, *p* < 0.001, *N* = 2,880, two-tailed Kendall rank correlation).

The relationship between odor liking and response time in odor naming was examined, with odor liking rated on a scale from 1 to 9, where 9 represents the highest level of liking. Similarly, to investigate the relationship between odor preference and naming response time, we conducted the Spearman and Kendall correlation analyses between liking ratings and response times. Participants showed faster naming responses for more liked odors, though this relationship was weaker than for familiarity, with response times ranging from 2.798 seconds for moderately disliked odors (level 4) to 1.602 s for highly liked odors (level 9), demonstrating that more pleasant odors were named slightly faster (Figure 5) (ρ = −0.081, *p* < 0.001, *N* = 2,880, two-tailed Spearman rank correlation). This weak negative relationship between odor liking and naming response time was consistently observed across correlation methods (τB = −0.057, *p* < 0.001, *N* = 2,880, two-tailed Kendall rank correlation).

### 3.5 Influence of the experience of a “tip-of-the-nose” state on odor naming

To investigate the effect of the TON feeling on the responses, correlation and Kruskal-Wallis tests were conducted. TON was rated between 1 and 9. Participants with higher tip-of-the-nose (TON) feelings showed better naming performance, with a clear progression from no response to incorrect to correct answers as TON scores increased, indicating that stronger TON sensations are associated with more successful odor naming attempts (ρ = 0.111, *p* < 0.001, *N* = 2,880, two-tailed Spearman rank correlation; τb = 0.093, *p* < 0.001, *N* = 2,880, two-tailed Kendall rank correlation). TON scores differed significantly across response accuracy groups, with participants who gave no response showing the lowest TON scores compared to those who attempted naming (*H* = 25.452, *p* < 0.001, df = 2, *N* = 2,880, Kruskal-Wallis test). *Post hoc* comparisons using Tukey’s test revealed that participants who provided no response had significantly lower TON scores than those who gave incorrect answers (*M* = −0.816, *p* < 0.001, Tukey HSD), while no significant differences were found between other group pairs (no response vs. correct: *M* = −0.428, *p* = 0.115; incorrect vs. correct: *M* = 0.388, *p* = 0.169, Tukey HSD).

### 3.6 Neural connections in odor naming: impact of cues

We employed Granger causality analysis to investigate whether lexical and color cues differentially shape bidirectional information flow and directed connectivity between brain regions during odor naming process. Granger causality analysis was performed on EEG data from 40 participants during lexical and color-cued odor naming tasks. Data were segmented into 4-s epochs beginning immediately after visual cue offset, when participants were instructed to think about the odor name. EEG signals were sampled at 500 Hz, with stationarity assessed using the Augmented Dickey-Fuller test (α = 0.05). Non-stationary time series were differenced up to two times to achieve stationarity. Vector autoregressive (VAR) modeling was applied with model order 10 (maxlag = 10), corresponding to 20 ms of historical information. A total of 992 directed connections (32 × 31 possible channel pairs) were analyzed for each participant.

The analysis revealed robust directed connectivity patterns across the participant group. Statistical significance was determined at *p* < 0.05 for individual connections, with robust connectivity defined as connections that were significant in more than 95% of participants. The analysis reveals distinct patterns of neural connectivity across different cueing paradigms during olfactory identification tasks.

**FIGURE 6:**
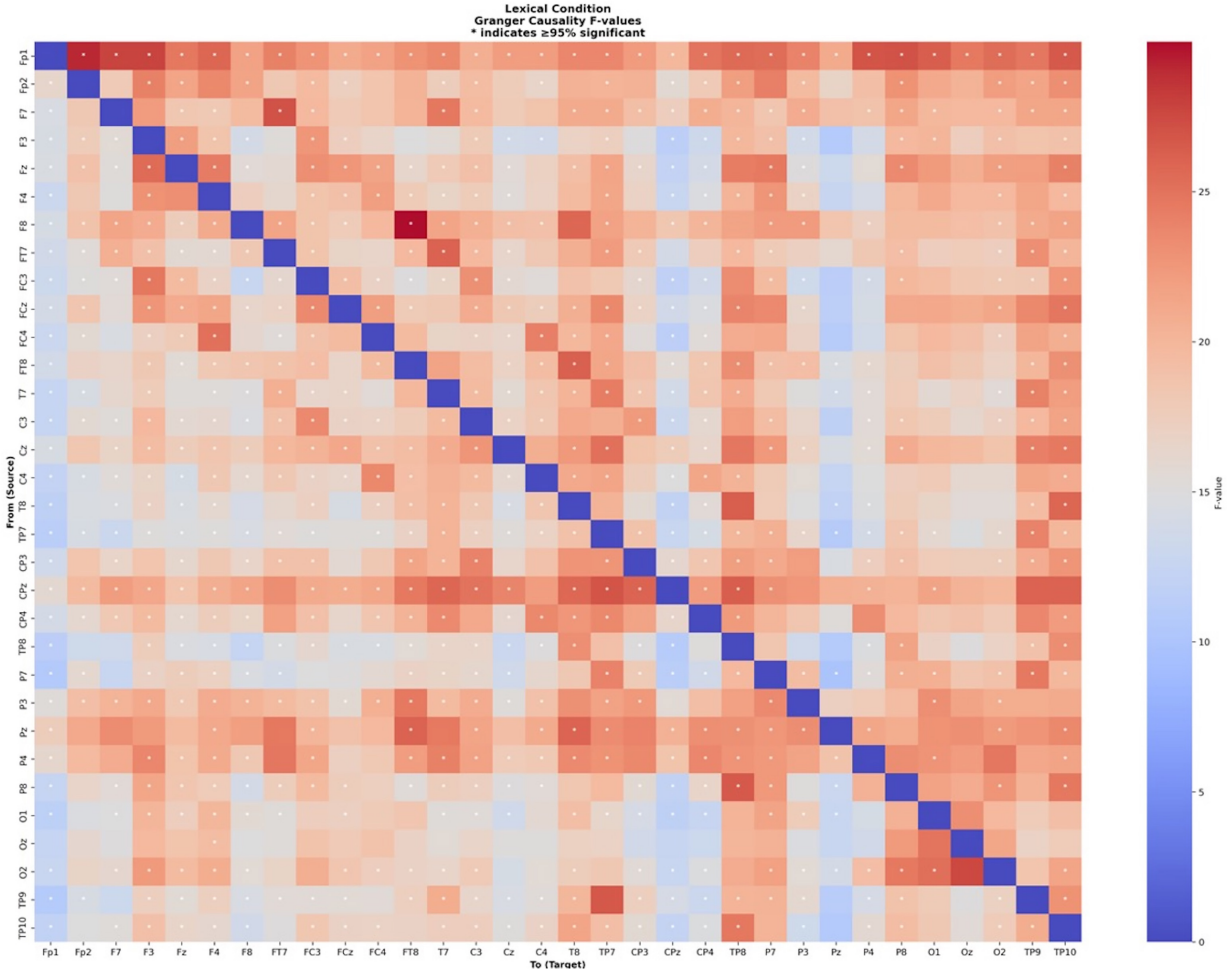
Granger causality connectivity matrix for odor naming after lexical cue. The heatmap displays directed functional connectivity (F-values) between all 32 EEG electrode pairs during lexical-cued odor naming. Colors represent connection strength (red = stronger, blue = weaker), with the diagonal showing self-connections (blue). Asterisks (*) mark connections significant in > 95% of participants. The matrix is organized by electrode position following the 10-20 system (Fp1, Fp2, F7, F3, etc.).

In the Granger causality analysis conducted on the odor naming task following lexical cues, 666 out of 992 possible directed connections met the robust significance criterion, corresponding to 67.1% of all connections (see Figure 6). The F-values for significant connections ranged from 9.90 to 29.99 (*M* = 18.69, SD = 4.23). The ten strongest connections were observed. The strongest connection shown from F8 to FT8 (*F* = 29.99, *p* = 0.0036). The second strongest connection demonstrated interhemispheric coordination between left and right prefrontal areas (Fp1 → Fp2, *F* = 29.08, *p* = 0.0087).

Strong medial prefrontal hub connectivity was evident through connections from Fp1 to left lateral frontal (F7), left dorsolateral prefrontal (F3), right parietal (P8, P4), and right temporal (TP10) regions, with F-values ranging from 26.59 to 27.87 (all *p* < 0.01).

Bilateral frontal-temporal circuits showed robust activation, with left hemisphere connections (F7 → FT7, *F* = 27.05) complementing the right hemisphere pattern. Cross-regional integration was demonstrated through central-parietal to left temporal-parietal connectivity (CPz → TP7, *F* = 26.94) and right parietal to right temporal-parietal flow (P8 → TP8, *F* = 26.58). Additionally, interhemispheric temporal-parietal coordination was observed from right to left temporal-parietal regions (TP9 → TP7, *F* = 26.6, *p* = 0.0045).

**FIGURE 7:**
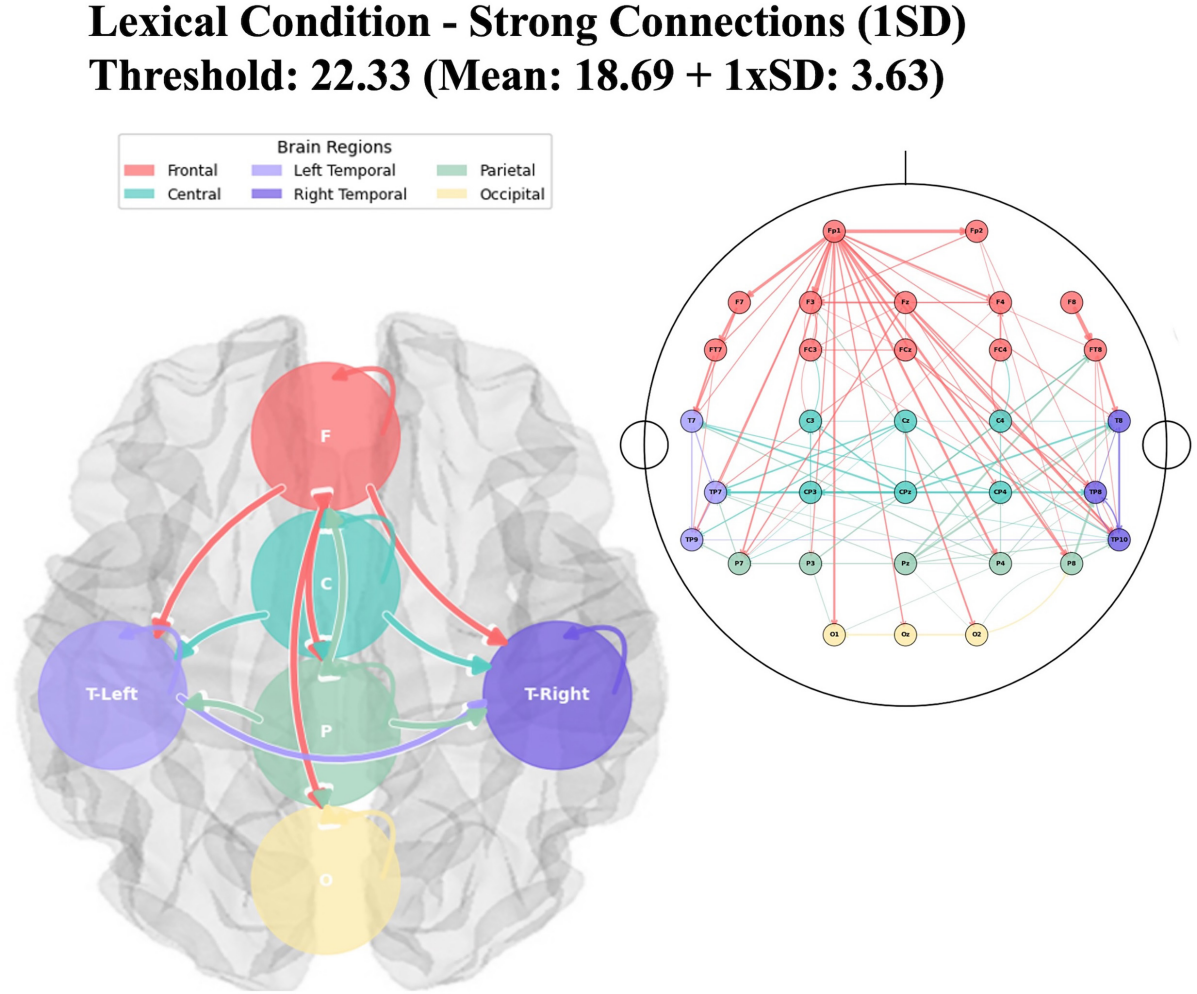
Regional brain network connectivity for odor naming after lexical cue. The figure displays the directional connectivity patterns between brain regions during odor naming following lexical cues, analyzed using Granger causality with a statistical threshold of 22.33 (Mean F-value: 18.69 + 1 × SD: 3.63). The right panel shows electrode-level connections mapped onto a standard 10–20 EEG montage, where nodes represent individual electrodes color-coded by brain region (Frontal: red, Central: teal, Left Temporal: light purple, Right Temporal: dark purple, Parietal: green, Occipital: yellow). The left panel presents the same connectivity data aggregated at the regional level, showing inter-regional causal relationships. Arrow thickness indicates the strength of Granger causality (*F*-values), with white borders enhancing visibility.

In the Granger causality connections between brain regions during the odor naming task after lexical cues, the lexical condition showed a mean F-value of 18.69 ± 3.63 with a threshold of 22.33 (see Figure 7). Frontal regions (F) exhibited connections to central, temporal, and parietal areas and showed reduced internal frontal connectivity compared to the color condition. The left temporal regions (T7, TP7) displayed increased outgoing connections to central and frontal areas. Central regions (C) maintained their connections with frontal areas and showed connectivity with temporal and parietal regions. Parietal regions (P) exhibited increased connections with frontal and central areas.

**FIGURE 8:**
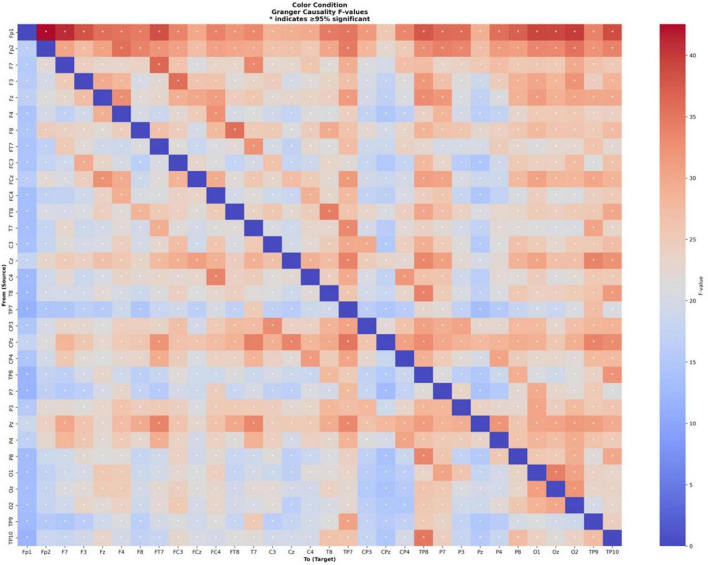
Granger causality connectivity matrix for odor naming after color cue. The heatmap displays directed functional connectivity (F-values) between all 32 EEG electrode pairs during color-cued odor naming. Colors represent connection strength (red = stronger, blue = weaker), with stronger overall connectivity compared to the lexical condition. Asterisks (*) mark connections significant in > 95% of participants. The matrix is organized by electrode position following the 10–20 system (Fp1, Fp2, F7, F3, etc.).

Granger causality analysis was performed on EEG data from 32 electrodes during the color cue condition, where participants viewed color cues related to odors and were instructed to think of the corresponding odor names. Out of 992 possible electrode pairs, 966 connections (97.4%) demonstrated significant Granger causality at the p < 0.05 level (see Figure 8). The F-values for significant connections in the color cue condition ranged from 11.61 to 42.54 (*M* = 23.66, SD = 5.18). The fifteen strongest Granger causality connections in the color cue condition revealed a different pattern of cortical information flow.

The strongest connection was observed between left and right prefrontal (Fp1 → Fp2, *F* = 42.54, *p* = 0.0046), indicating robust interhemispheric coordination. This was followed by strong prefrontal to left lateral frontal connectivity (Fp1 → F7, *F* = 41.15, *p* = 0.0117). Prominent connections emerged from prefrontal to occipital regions, including Fp1 → O2 (F = 39.92), Fp1 → O1 (F = 39.28), and Fp1 → Oz (*F* = 39.20).

Fp1 emerged as the dominant source hub, showing strong outgoing connections to temporal poles (TP10, TP8), frontal (F3), frontal-temporal (FT7) and parietal (P8, P7, P3, P4) regions, with F-values ranging from 35.84 to 38.60 (all *p* < 0.013). Left hemisphere frontal-temporal coordination remained prominent (F7 → FT7, *F* = 36.40, *p* = 0.0064).

**FIGURE 9:**
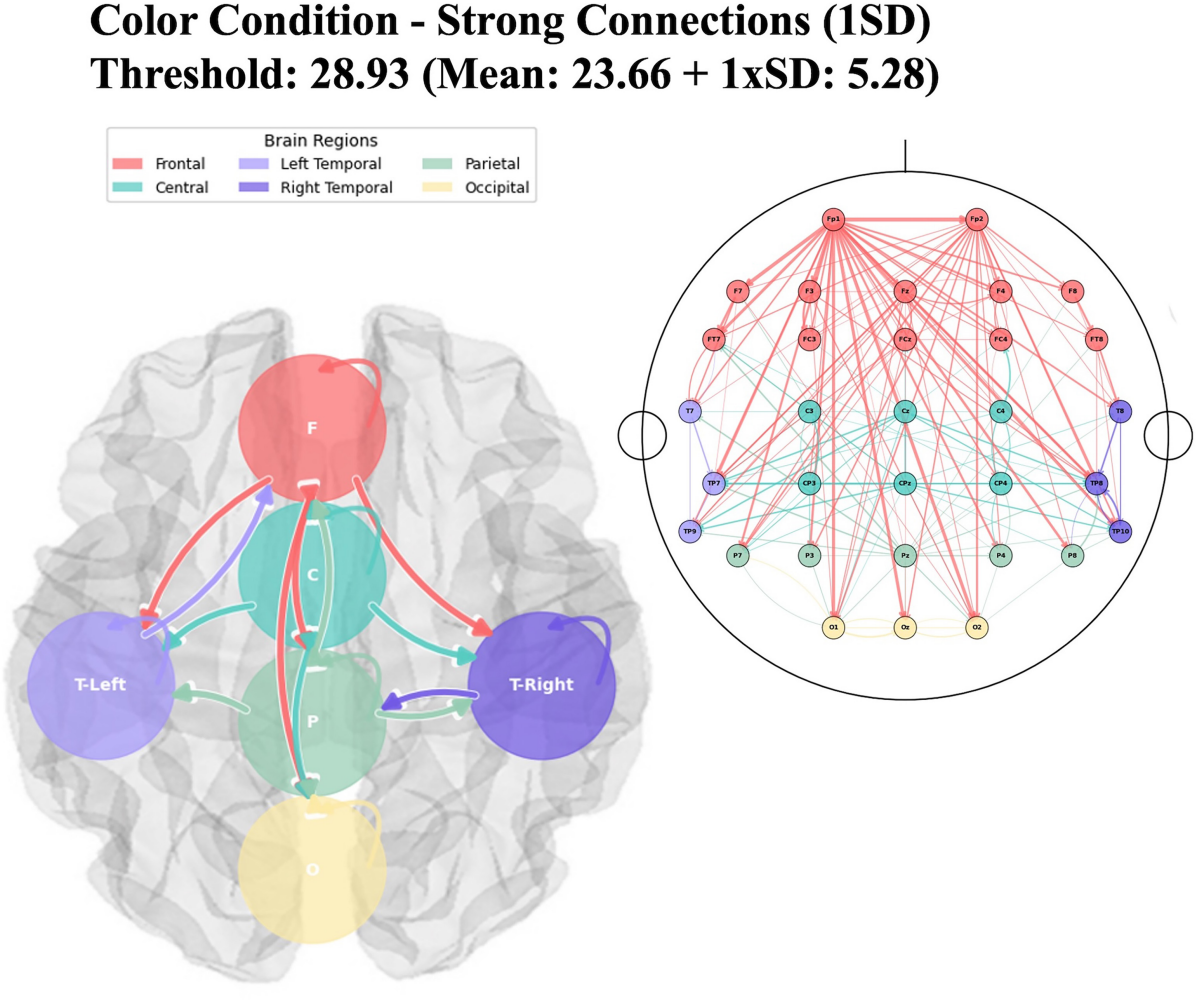
Regional brain network connectivity for odor naming after color cue. The figure displays the directional connectivity patterns between brain regions during odor naming following color cues, analyzed using Granger causality with a statistical threshold of 28.93 (Mean *F*-value: 23.66 + 1 × SD: 5.28). The right panel shows electrode-level connections mapped onto a standard 10–20 EEG montage, where nodes represent individual electrodes color-coded by brain region (Frontal: red, Central: teal, Left Temporal: light purple, Right Temporal: dark purple, Parietal: green, Occipital: yellow). The left panel presents the same connectivity data aggregated at the regional level, showing inter-regional causal relationships. Arrow thickness indicates the strength of Granger causality (*F*-values), with white borders enhancing visibility.

In the Granger causality connections between brain regions during the odor naming task after color cues, the color condition showed a mean F-value of 23.66 ± 5.28 and a threshold of 28.93 (see Figure 9). Frontal regions (F) demonstrated extensive internal frontal-frontal connectivity and projections to central, temporal, and occipital areas. Central regions (C) showed bidirectional connectivity with frontal areas and connections to temporal and parietal regions. Bilateral temporal connectivity was observed between left and right hemisphere temporal regions, with projections to central areas. Strong connections from frontal, central, and temporal areas were directed toward the occipital regions (O).

**FIGURE 10:**
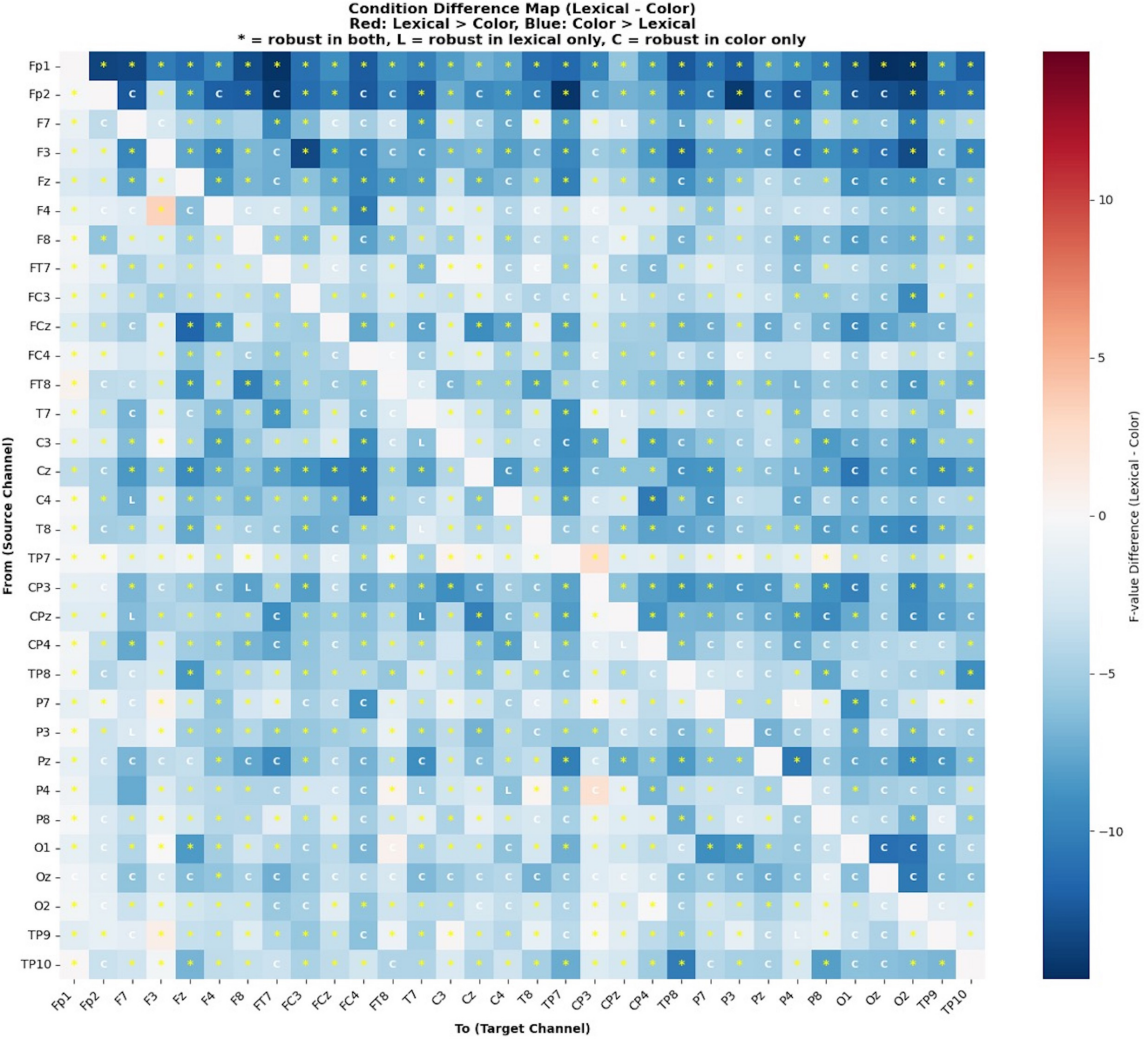
Directional connectivity differences between odor naming after lexical and color conditions based on granger causality analysis.

During the odor naming task, differences in neural connections were examined based on lexical and color priming conditions (see Figure 10). The extremely high proportion of significant connections (97.4%) indicates more extensive cortical engagement during color-cued olfactory naming compared to the lexical condition (67.1%). F-values in the color cue condition were substantially stronger, ranging from 11.61 to 42.54 (*M* = 23.66) compared to the lexical condition range of 9.90 to 29.99 (*M* = 18.69). The peak connectivity strength was 42% higher in the color cue condition (*F* = 42.54) than in the lexical condition (*F* = 29.99).

A marked difference in visual system activation was observed between the color cue and lexical cue conditions. The difference matrix revealed an asymmetric pattern, where the advantages of color cues were both more numerous and of greater magnitude compared to lexical advantages. Color-dominant differences frequently exceeded -5 F-units, with some connections approaching -10 F-units, whereas lexical-dominant differences rarely exceeded + 5 F-units. Robust differences (indicated by asterisks) predominantly favored the color cue condition, particularly along the frontal-posterior pathways. Markers labeled “C” indicate robust connections specific to the color cue condition but not present in the lexical condition, while “L” markers denote lexical-specific robust connections. The distribution pattern showed a marked asymmetry, with significantly more “C” than “L” markers, confirming that color cue processing engages additional neural pathways not recruited during lexical processing.

Unlike the lexical condition, the color condition revealed three strong frontal-occipital connections (F → O1, Oz, O2). Despite differences in cue type, the F7 → FT7 connection remained consistently active across conditions.

The color cue condition exhibited more extensive posterior cortical interaction, with strong connections from the medial prefrontal regions to temporal (TP10, TP8), parietal (P8, P7, P3, P4), and occipital (O2, O1, Oz) regions. In contrast, the lexical condition demonstrated more focused connectivity, primarily involving frontal-temporal and frontal-parietal circuits, with limited involvement of the occipital region.

Both conditions showed bilateral cortical interaction, but with different distributions. The lexical condition showed balanced frontal activation, with the strongest connection from right lateral frontal to right fronto-temporal regions (F8 → FT8, *F* = 29.99). The color cue condition demonstrated enhanced prefrontal coordination, with the strongest connection observed in the interhemispheric prefrontal region (Fp1 → Fp2, *F* = 42.54). The connectivity patterns suggest that the conditions involve different cognitive processing demands. Conversely, lexical priming appears to recruit more focused and specific neural pathways.

## 4 Discussion and conclusion

### 4.1 Effect of cue modality on odor identification performance

This study revealed the effects of different types of cues on the odor naming process. When examining the impact of lexical and color cues on odor naming accuracy, lexical cues led to significantly higher correct identification performance.

The observed difference in naming accuracy highlights the important role of semantic information in odor identification (Stevenson and Wilson, 2007). This may be understood in light of dual coding theory (Paivio, 1991), which proposes that information encoded verbally tends to provide stronger and more accessible retrieval cues compared to visual features. Verbal information often engages abstract conceptual networks, whereas visual cues such as shapes and colors are typically linked to more concrete object representations. Lexical cues improve odor naming accuracy by activating rich semantic memories and specialized neural networks involved in language processing (Herz, 2003; Olofsson et al., 2014). Consistent with this, our findings indicate that odor memory is more closely connected to verbal memory than to color perception. This pattern aligns with Tulving and Thomson’s encoding specificity principle, which emphasizes that memory retrieval is more effective when the retrieval context matches the original encoding context (Tulving and Thomson, 1973). Since odors are usually encoded alongside verbal labels, lexical cues provide a more direct route to these memories compared to color cues. However, despite the clear advantage of lexical cues in accuracy, our results reveal an unexpected dissociation: lexical cues did not lead to faster naming. This suggests that different cognitive mechanisms may underlie accuracy and speed during odor processing.

Given these findings, it is important to clarify our methodological approach to odor identification assessment. In our study, we considered odor naming performance as an indicator of odor identification under the different types of cues. Cain et al. (1998) emphasized that odor identification involves complex aspects of semantic memory and highlighted the interactive nature of perceptual and verbal processes. In the literature, odor naming performance is frequently employed as a measure of odor identification. For example, Cameron et al. (2016) assessed odor identification ability using a task that required naming both common and rare odors, and found that participants attempted to generate names for most of them. Similarly, Fusari and Ballesteros (2008) evaluated odor identification performance with 64 natural odors to examine the effects of age and gender, using naming success as the primary measure. Moss et al. (2016) collected normative data for 200 odors to be used in olfactory memory experiments, considering the odors’ nameability as a key evaluation criterion. Nonetheless, it should be recognized that dissociations may exist between these two processes. Cleary et al. (2010), for instance, developed the “recognition without identification” paradigm, demonstrating that participants could perceptually recognize odors without being able to name them. In our study, by examining the TON phenomenon, we also evaluated the behavioral reflections of the effect of the dissociation between perceptual recognition and verbal access.

A limitation of our study is the absence of a control condition, such as non-informative or incongruent visual cues, to directly assess the effect of visual cues on odor identification, because we focused on congruent different types of cues. Studies in the literature consistently show that semantically congruent visual cues can significantly enhance odor identification performance. Demattè et al. (2009) reported that semantically congruent visual stimuli improved odor identification accuracy. Gottfried and Dolan (2003) demonstrated that congruent visual-olfactory pairs enhanced odor perception performance and increased neural activity in the anterior hippocampus and rostromedial orbitofrontal cortex. Similarly, Olofsson et al. (2014) investigated olfactory-language integration and examined the effects of congruent and incongruent cues. According to their findings, semantically incongruent pairs are processed more slowly and less accurately. EEG measurements indicated that the N400 response, a marker of semantic incongruity, was more localized to posterior electrode sites. In light of these findings, although we cannot conclusively determine whether the visual cues used in our study truly enhance odor naming performance compared to unrelated visual stimuli, we aim to make a significant contribution to the field by comparing the effects of different types of congruent visual cues. In future studies, it is recommended to include incongruent or random visual stimuli as control conditions to more clearly determine the specific contribution of semantically relevant visual cues to odor naming.

### 4.2 Influence of familiarity and preference on odor identification

We observed strong negative correlations between both odor familiarity and odor liking with response time, reinforcing the influence of these factors on olfactory processing efficiency (Herz, 2016; Larsson, 1997; Wilson and Stevenson, 2003). Familiar odors benefit from reinforced memory representations developed through repeated exposure, enabling faster identification (Wilson and Stevenson, 2003). Similarly, odors with higher hedonic value tend to be processed more quickly, reflecting the attentional priority and emotional salience these stimuli hold (Herz, 2016). Together, these findings suggest that both familiarity and liking to facilitate more rapid odor recognition, likely through complementary cognitive and affective mechanisms.

### 4.3 Tip-of-the-nose phenomenon in olfactory naming

The significant relationship we detected between response types and the TON phenomenon in our study reveals the critical effect of this metacognitive process on odor perception. The positive correlation found between TON scores and response status (correct, incorrect, and no response), particularly the significant difference between the no-response group and the incorrect-answer group, suggests that TON experience reflects partial access to memory. These findings corroborate Jönsson and Olsson (2003) findings that individuals experiencing TON can suggest alternative names that have semantic connections with the target odor, even though they cannot produce the completely correct response (Jönsson and Olsson, 2003). This situation confirms the possibility of partial access to the memory system and validates the mediating role of the TON phenomenon in the odor recognition process.

### 4.4 Neural connectivity underlying cue-based odor naming

One of the longstanding challenges in olfactory processing is understanding how the human brain integrates different cue modalities during odor recognition and whether these processes lead to distinct neural network organizations. Previous research has shown that visual cues can aid odor perception (Gottfried and Dolan, 2003) and that integrating olfactory and linguistic information requires specialized neural substrates (Olofsson et al., 2014). However, limited evidence exists regarding modality-specific differences in neural networks involved in odor recognition and their temporal dynamics. In this study, findings based on Granger causality analysis reveal that lexical and color cues produce markedly different effects on odor recognition processes, indicating the engagement of modality-specific neural strategies. Our results strongly suggest that the human brain exhibits either selective (focal) or widespread (diffuse) activation patterns in response to different types of cues, and that these differences are closely linked to cognitive efficiency.

The selective connectivity patterns observed under the lexical cue condition suggest that word-based cues activate specific neural networks. Interestingly, the strongest connection was found between the right lateral frontal region and the right fronto-temporal area, indicating a shift from the traditionally left-lateralized patterns of language processing. This finding implies that right-hemispheric structures also significantly contribute to the interaction between olfactory processing and language systems. It further supports the idea that the brain’s odor-language integration system (Olofsson et al., 2014) shows modality-specific lateralization characteristics.

The strong connections from the medial prefrontal hub to bilateral temporal and parietal regions suggest that lexical cues help enable direct semantic access. This pattern matches previous findings showing the important role of temporal lobe structures in connecting olfactory perceptions to verbal information (Savic et al., 2000). The strong activation of bilateral fronto-temporal circuits further suggests that odor-language integration involves specialized neural substrates and aligns with evidence emphasizing the role of prefrontal regions in coordinating advanced olfactory processing (Howard et al., 2009).

A notable finding under the lexical cue condition is the strong emergence of interhemispheric temporal-parietal coordination. This suggests that olfactory-lexical integration is not limited to a single hemisphere but instead involves bilateral organization. In line with Dalal et al. (2020), demonstrating that bilateral processing is essential for odor perception and that interhemispheric coordination maintains perceptual coherence in olfactory processing, our findings suggest that such bilateral interactions are also crucial when combining olfactory and linguistic information. Additionally, the bilateral connectivity observed between frontal regions suggests that these areas assume a regulatory role in coordinating this complex interhemispheric process.

In the color cue condition, a much more widespread pattern of connectivity was observed. This suggests that visual-olfactory integration requires broader cortical mobilization compared to lexical cues. Notably, the strongest connection observed was interhemispheric prefrontal coordination, along with projections to occipital areas. These findings suggest that cross-modal integration needs active coordination between the visual cortex and semantic processing regions. This aligns with the findings of Gottfried and Dolan (2003) who demonstrated that semantically congruent visual cues enhance olfactory perception.

The focused frontal coordination observed in the lexical cue condition is further explained by Olofsson et al. (2013, 2014) who identified involvement of the right orbitofrontal cortex and anterior temporal lobe through ERP and fMRI methods. In contrast, the more distributed and widespread prefrontal activation observed under color cues suggests that this process likely occurs via an indirect semantic route. Color cues may have triggered multiple associations, making it harder to identify the target odor object and resulting in lower behavioral accuracy. This ambiguity might have required the engagement of cognitive control systems to select the correct response. Consequently, an increase in connectivity between control regions such as the dorsolateral prefrontal cortex (DLPFC) and semantic networks may have been observed under color cue conditions.

On the otherhand color–odor associations can established through verbal descriptors (e.g., “strawberry” odor → “strawberry” label → red color), reflecting a multi-step transformation process: color → object → odor → name. This pathway demands substantial neural resources. As demonstrated by Seubert et al. (2017) the piriform cortex processes affective visual information prior to olfactory perception, supported by a network involving the orbitofrontal cortex, amygdala, and hippocampus. Additionally, multisensory facilitation enhances odor object processing in the primary olfactory cortex (Porada et al., 2019), and transmodal regions such as the temporal pole and anterior temporal lobe play a crucial role in integrating information from different modalities (Mesulam et al., 2013; Olofsson et al., 2013).

Psycholinguistic factors such as word frequency have also been shown to critically affect odor naming performance (Speed and Majid, 2019). In this context, lexical cues offer a more direct and efficient route to semantic access, whereas color cues entail a more cognitively demanding transformation. This distinction is evident in our findings, where the connection strength and density differ significantly between lexical and color cue conditions. Notably, even congruent color cues require high cortical connectivity, indicating that this modality does not facilitate direct verbal access but instead relies on a multi-stage semantic conversion. This cognitive demand arises from the need to link perceptual input with semantic representations: upon perceiving a color cue, individuals must activate a related object category (e.g., “strawberry”), retrieve the associated odor representation, and access the corresponding label. These associations are shaped by semantic consistency, learning experiences, and cultural context, and necessitate a source-based mapping approach (Demattè et al., 2006).

Canini et al. (2016) observed increased activation in the left inferior frontal gyrus and left caudate nucleus during semantic processing in their functional neuroimaging study examining semantic interference processes. The dense inter-frontal connectivity network we detected in the color cue condition overlaps with these frontal cortex findings. The strong frontal-occipital connections we identified reveal that transforming visual stimuli into semantic representations is a highly complex process. This connectivity pattern also aligns with Lwin et al. (2016) findings that cross-modal integration requires coordinated activity between the visual cortex and semantic processing centers. It seems that color cues demand the engagement of a much broader cortical network compared to lexical cues.

The findings of this study show that cue modality significantly affects performance and cognitive load in olfactory evaluation tasks. Compared to letter cues, color cues require more cross-modal integration, visual-semantic processing, and executive functions. This is an important difference to consider in neuropsychological assessment and rehab. Our study highlights the complexity of cross-modal semantic access and reveals that optimal cognitive performance is not always linked to wider cortical activation, but rather to efficient and selective neural network engagement. Although color cues are aligned, they show lower behavioral performance and higher connectivity. This indicates that increased brain activation does not always lead to better performance, supporting the idea of network efficiency. The more focused temporal-frontal coordination seen under the lexical condition helps identification by allowing direct semantic access, while in the color condition, this process involves a more effortful semantic route.

It is essential to acknowledge the methodological limitations of the Granger Causality analyses conducted in this study. GC analyses are susceptible to structured confounds such as shared autocorrelation, volume conduction, and filtering effects. Although we employed several techniques to improve statistical robustness, such as outlier correction, winsorizing, group-level aggregation, and FDR correction, these methods do not completely eliminate potential structural confounds. In particular, when similar autocorrelated structures exist across participants, group-level aggregation may not completely remove these effects. Future studies should incorporate control analyses such as the time-reversal test (which involves reversing the temporal order of each signal) to further enhance the reliability of GC findings.

In conclusion, this study examined behavioral and neural responses to odor-naming performance using color and letter cues. Our findings showed that lexical cues resulted in higher accuracy compared to color cues, with some odors receiving no correct responses under color conditions. Granger causality analysis revealed distinct neural strategies for different cue types. Lexical cues activated focused frontal-temporal circuits that enable direct semantic access, while color cues required broader cortical network activation through an indirect semantic pathway. This difference was indicated by higher F-values in the color condition compared to the lexical condition. A key finding is that optimal cognitive performance is associated with efficient and selective neural network engagement rather than broader cortical activation. Despite being congruent, color cues showed lower behavioral performance and higher connectivity, demonstrating that increased brain activation does not always lead to better performance. These findings highlight the critical role of cue modality in olfactory assessments. Lexical cues evaluate direct lexical access, while color cues assess comprehensive cross-modal semantic processing.

Our findings indicate that the odor naming process has a multi-factor structure with the effects of both cognitive and sensory factors. Our results support that the odor perception and naming process is not limited to only the direct function of the olfactory system, and that our past experiences and evaluations also make important contributions to this process.

This study has limitations that should be taken into consideration. First, the absence of a universally accepted “gold standard” for odor recognition accuracy raises concerns about the reliability of the findings. In this study, responses were classified as correct only if the odor name was accurately identified; all other responses were considered incorrect.

Additionally, the study used synthetic odors presented under controlled laboratory conditions. While this allows for experimental control, it may not fully capture the complexity of real-world olfactory experiences, which are influenced by dynamic environmental factors. Moreover, the influence of other sensory modalities such as auditory and tactile cues was not considered, potentially overlooking important aspects of multisensory integration in odor perception.

From a methodological perspective, although EEG provides high temporal resolution, it lacks the spatial precision of techniques such as fMRI. Employing fMRI could offer more detailed insights into the neural processing of olfactory stimuli.

Future research should consider using more ecologically valid odor sources and investigate the interaction of olfaction with multiple sensory cues to develop a more comprehensive understanding of odor perception. Experimental designs should also consider the environmental context and variability. Ultimately, combining the temporal resolution of EEG with the spatial resolution of fMRI may provide a more comprehensive understanding of the neural mechanisms underlying odor recognition.

## Data Availability

The raw data supporting the conclusions of this article will be made available by the authors, without undue reservation.
