# Supplementary material for: Cues for odor naming affect performance and brain connectivity
Source: Front Hum Neurosci. 2025 Dec 2;19:1671670. doi: 10.3389/fnhum.2025.1671670 (PMC12706689; doi:10.3389/fnhum.2025.1671670)
Supplement: Supplementary file 1 [file Data_Sheet_1.docx]

Supplementary Material

· The odor is intense (1-9)

· I am familiar with the odor (1-9)

· I like the odor (1-9)

· I know the name of the odor, it's on the tip of my tongue (1-9)

· I know the name of the odor (1-9)

· I am sure of the name of the odor (1-9)

· Can you name a similar odor? (1-9)

· What is a similar odor? (Open-ended question)

· Can you provide a general category for the odor? (Open-ended question)

· Can you name an object that the odor might have come from? (Open-ended question)

· Can you name a place where the odor might have come from? (Open-ended question)

· What is the effect of the cue on naming? (1-9)

*Rating Scale Items (1-9 scale; 1 = not at all, 9 = extremely)*
